# MRI predictors of clinical success in MR-guided focused ultrasound (MRgFUS) treatments of uterine fibroids: results from a single center

**DOI:** 10.1186/2050-5736-3-S1-O99

**Published:** 2015-06-30

**Authors:** Irene Mindjuk

**Affiliations:** 1 Amper Kliniken AG, Dachau, Germany

## Background/introduction

To assess the technical and clinical results of MRgFUS treatment and factors affecting clinical treatment success.

## Methods

252 women (mean age, 42.1 ± 6.9 years) with uterine fibroids underwent MRgFUS treatment using an ExAblate 2100 system (Insightec Ltd., Israel). All patients underwent MRI screening before treatment. Results were evaluated with respect to post-treatment non-perfused volume (NPV), symptom severity score (SSS), reintervention rate, pregnancy and safety data.

## Results and conclusions

NPV ratio was significantly higher in fibroids characterized by low signal intensity in contrast-enhanced T1-weighted fat saturated MRI images and in fibroids that are distant from spine (>3cm). NPV ratio was lower in fibroids with septations, with subserosal component and in skin-distant fibroids (p< 0.001). NPV ratio was in high correlation with clinical success: NPV of more than 80% resulted clinical success in more than 80% of patients. Reintervention rate was 12.7% (mean follow-up time, 19.4±8 months; range, 3-38). Expulsion of fibroids (22%) significantly correlated with a high clinical success rate. No severe adverse events were reported.

